# Measuring evidence-based practice knowledge and skills in occupational therapy—a brief instrument

**DOI:** 10.1186/s12909-015-0475-2

**Published:** 2015-10-30

**Authors:** Helen Buchanan, Jennifer Jelsma, Nandi Siegfried

**Affiliations:** Department of Health & Rehabilitation Sciences, University of Cape Town, F45 Old Groote Schuur Hospital Building, Observatory, 7925 Cape Town, South Africa; Independent Clinical Epidemiologist, Cape Town, South Africa

**Keywords:** Occupational therapy, Evidence-based Practice, Knowledge, Skills, Adapted Fresno Test of Competence in Evidence-based Practice, Evaluation, Instrument, Psychometric, Reliability, Responsiveness

## Abstract

**Background:**

Valid and reliable instruments are required to measure the effect of educational interventions to improve evidence-based practice (EBP) knowledge and skills in occupational therapy. The aims of this paper are to: 1) describe amendments to the Adapted Fresno Test of Competence in EBP (AFT), and 2) report the psychometric properties of the modified instrument when used with South African occupational therapists.

**Methods:**

The clinical utility of the AFT was evaluated for use with South African occupational therapists and modifications made. The modified AFT was used in two studies to assess its reliability and validity. In Study 1 a convenience sample of 26 occupational therapists in private practice or government-funded health facilities in a South African province were recruited to complete the modified AFT on two occasions 1 week apart. Completed questionnaires were scored independently by two raters. Inter-rater, test-retest reliability and internal consistency were determined. Study 2 was a pragmatic randomised controlled trial involving occupational therapists in four Western Cape Department of Health district municipalities (*n* = 58). Therapists were randomised in matched pairs to one of two educational interventions (interactive or didactic), and completed the modified AFT at baseline and 12 weeks after the intervention. An intention-to-treat analysis was performed. Data were not normally distributed, thus non-parametric statistics were used.

**Results:**

In Study 1, 21 of 26 participants completed the questionnaire twice. Test-retest (ICC = 0.95, 95 % CI = 0.88–0.98) and inter-rater reliability (Time 1: ICC = 0.995, 95 % CI = 0.99–0.998; Time 2: ICC = 0.99, 95 % CI = 0.97–0.995) were excellent for total scores. Internal consistency based on time 1 scores was satisfactory (α = 0.70). In Study 2, 28 participants received an interactive educational intervention and completed the modified AFT at baseline and 12 weeks later. Median total SAFT scores increased significantly from baseline to 12-weeks (*Z* = −4.078, *p* < 0.001) with a moderate effect size (*r* = 0.55).

**Conclusion:**

The modified AFT has demonstrated validity for detecting differences in EBP knowledge between two groups. It also has excellent test-retest and inter-rater reliability. The instrument is recommended for contexts where EBP is an emerging approach and time is at a premium.

**Trial registration:**

Pan African Controlled Trials Register PACTR201201000346141. Registered 31 January 2012.

Clinical Trials NCT01512823. Registered 1 February 2012.

South African National Clinical Trial Register DOH2710093067. Registered 27 October 2009.

## Background

The obligation for health professionals to base their practice on solid evidence requires a certain level of knowledge and skills in evidence-based practice (EBP). Educational interventions are commonly used to equip health professionals with the tools for EBP, but valid and reliable instruments are required to measure the effects of such interventions in improving knowledge and skills [1, 2]. At the time of this study (2008), no research had been conducted into the effects of educational interventions with occupational therapists in South Africa, and since then only one study has been published [3]. A national survey of this group revealed positive perceptions towards EBP but poor confidence in EBP skills with a sizeable proportion of respondents (31 %) attributing this to their limited knowledge and skills [4]. Few had attended EBP training (25 %) and relied strongly on their clinical experience rather than research literature. Their limited success in finding and applying evidence may have been due to reported barriers such as time, knowledge, and convenient access to evidence sources. The survey findings suggested a need for further education, and a randomised controlled trial (RCT) was planned to test the effects of two educational interventions in this group and context.

To identify existing instruments to measure changes in EBP knowledge and skills specifically within occupational therapy, searches were conducted in Pubmed and EBSCOHost (Africa-Wide: Information, CINAHL, ERIC, Health Source: Nursing/academic edition, MEDLINE, PsycARTICLES and PsycINFO) from their inception until May 2008. The search terms used were:

(“occupational therapy” OR “occupational therapy practice” OR OT) AND (tool OR survey OR instrument OR test OR measure OR scale OR questionnaire) AND (“evidence-based practice” OR “evidence based practice” OR EBP OR “evidence-based-medicine” OR evidence-based) AND (knowledge OR awareness OR skills OR attitudes OR perceptions OR behaviour OR practice OR ability OR uptake OR implementation OR “research use” OR “research utilisation” OR “research utilization”).

Terms were searched individually and results sets combined. No limits were set and no attempt was made to identify unpublished materials. Reference lists of included studies were checked for articles that may have been missed. Articles identified in the searches were screened and those that included instruments measuring EBP knowledge and/or skills in occupational therapists were selected and examined to identify those with evaluative properties.

The search revealed a paucity of instruments for measuring EBP educational outcomes (*n* = 7). Contact with two occupational therapists involved in EBP research revealed one further instrument [5]. Of the eight identified instruments (see summary in Table 1), two, the Adapted Fresno Test of Competence in EBP (AFT) [6] and the modified Knowledge, Attitude and Behavior (KAB) questionnaire [5], were evaluative and had been used in studies involving occupational therapists.

**Table 1:** Summary of articles identified in the search (*n* = 10)

Author (year)	Original source (date)/Name of instrument (where applicable)	Location	Participants	Study design	EBP attributes measured	Instrument structure	Purpose of instrument
Bennett, Tooth, McKenna, Rodger, Strong, Ziviani, Mickan and Gibson [29]	Adapted from McColl, Smith, White and Field [30]	Australia	Members of OT Australia (*n* = 649)	Cross-sectional	Confidence in skills related to the acquire and appraise steps in the EBP process	Self-report with 4 sections; 5 questions with a 5-point rating scale for this attribute	Descriptive
Dysart & Tomlin [31]	NA	United States	Random sample of members of the American Occupational Therapy Association (*n* = 209)	Cross-sectional	Skills	Self-report with 3 sections containing dichotomous, 4-point scale, 5-point Likert scale and open-ended responses	Descriptive
MacDermid, Solomon, Law, Russell and Stratford [5]	Modified knowledge, attitude and behavior questionnaire [15]	Canada	Physical and occupational therapists (*n* = 144)	Randomised controlled trial	Knowledge	Subjective (self-report) and objective items using mainly 5-point scales, dichotomous scales and numerical items	Evaluative
McCluskey [32]	Adapted from Upton and Lewis [33]	New South Wales, Australia	OTs attending an EBP workshop (*n* = 67)	Cross-sectional	Knowledge and skills related to the ask, acquire, appraise and apply steps	Self-report containing 7 items for knowledge and skills respectively; 3-point rating scale	Descriptive
McCluskey and Lovarini [6]	Based on Bennett, Tooth, McKenna, Rodger, Strong, Ziviani, Mickan and Gibson [29] and Upton and Lewis [33]	Australia	OTs attending an EBP workshop (*n* = 114)	Before-after study	Knowledge and perceived skill in the ask, acquire, appraise and apply steps	Self-report with 17 items in Section 3 measuring these attributes; mix of objective and self-report items; 3-point rating scale	Descriptive
McCluskey and Lovarini [6]	Adapted Fresno Test of Competence in EBP	Australia	OTs attending an EBP workshop (*n* = 114)	Before-after study	EBP knowledge and skills – developing a PICO question, searching for evidence and appraising evidence	Objective assessment with 7 open-ended questions based on clinical scenarios; scored with a grading rubric (scoring range: 0 to 156)	Evaluative
Pain, Hagler and Warren [34]	Edmonton Research Orientation Survey (EROS)	Province in the West of Canada	OTs in 2 large urban and 2 rural/small urban areas (*n* = 58)	Cross-sectional	Self-rated knowledge of research concepts	38 items rated on a 5-point Likert scale; an overall score and sub-scale scores are calculated	Descriptive
Upton and Lewis [33]		Wales, United Kingdom	Podiatrists (*n* = 38), OTs (*n* = 84), physiotherapists (*n* = 135) and speech therapists (*n* = 38)	Cross-sectional	Perceived knowledge and skills of EBP and its individual steps	Self-report with 5 sections using varying question formats (visual analogue scales, semantic differentials, 5-point scales and space for comments)	Descriptive

The AFT was adapted for occupational therapy from the Fresno Test of Competence in Evidence-based Medicine [7] and is an objective measure of knowledge and skills in the EBP process [6]. The original 12 questions in the Fresno Test were reduced to seven by removing questions ‘about diagnosis and more complex statistical calculations’ [6: 120]. Six clinical scenarios were developed to provide three versions of the test (Versions 1 to 3) so the effects of EBP training can be evaluated at three time points. All items require open-ended responses that are judged as ‘excellent’, ‘strong’ or ‘limited’ according to criteria in a scoring rubric. The maximum score is 156. The AFT is advocated as ‘most useful for demonstrating change in novice learners of EBP’ [6: 125].

Additional information on the psychometric properties of the AFT were obtained from the developer (Dr A. McCluskey) at the time of the study, and have since been published [8]. Inter-rater reliability (IRR) for total AFT scores is reported as excellent for Versions 1 (ICC = 0.96; 95 % CI = 0.83–0.99) and 2 (ICC = 0.91; 95 % CI = 0.69–0.98), and internal consistency moderate for Version 1 (α = 0.74). The AFT was responsive to change in a cohort of occupational therapists attending a two-day educational workshop with a mean change score of 20.6 of 156 points and a large effect size for the total score (*d* = 0.8; 95 % CI: 15.6–25.5) [8].

The AFT has since been used in several occupational therapy studies [9–11]. Tilson [12] assessed the original Fresno Test and the AFT for physiotherapists but found both unsuitable as they only assess steps 1 to 3 of the EBP process. Stressing the importance of including an assessment of steps 4 and 5, she modified the original Fresno Test to make it applicable to physiotherapists and added two questions to assess the 5-step EBP process more comprehensively [12]. This modification of the Fresno Test has been reported to have excellent IRR (ICC = 0.91, 95 % CI: 0.87–0.94) and intra-rater reliability (rater 1: ICC = 0.95, 95 % CI: 0.90–0.98; rater 2: ICC = 0.96, 95 % CI: 0.90–0.98), acceptable internal consistency (α = 0.78) and content and discriminative validity [12]. A limitation of the original Fresno Test [12, 13] as well as the AFT [14] and modified Fresno for physiotherapists [12] is the time required for completion and scoring. To overcome these limitations, the Fresno was customised for entry level health profession students by revising its content and changing the response options to tick box and true/false formats rather than the original open-ended questions [14]. The new instrument, the Knowledge of Research Evidence Competencies (K-REC) instrument, has moderate to excellent test-retest (ICC = 0.88) and IRR (ICC = 0.97) for total scores, and was able to discriminate between students with no previous EBP exposure and those with some exposure. A Dutch translation and evaluation of the content of the Fresno Test similarly found it too difficult for undergraduate allied health science students and replaced the open ended questions with multiple choice, yes/no and short answer formats to provide more structure [13].

The KAB questionnaire was developed for medical students in Hong Kong [15] and subsequently modified for a study with Canadian rehabilitation therapists, including occupational therapists, by changing the medical terminology [5]. The questionnaire contains 53 items with a mix of answer options including 6-point, 5-point and dichotomous scales, fill in the blank, tick boxes and open-ended responses. Factor analysis identified four separate factors related to EBP: knowledge, attitudes, personal application and use, and future use. The knowledge factor contains five items rated on a 6-point scale. The KAB questionnaire has shown an ability to measure change in EBP knowledge in second year medical students eight months after attending six EBP modules (*d* = 0.33, *p* < 0.01), but the other three factors failed to show a significant change in scores [15].

The AFT was assessed as more suitable than the KAB questionnaire for measuring EBP knowledge and skills in occupational therapists as it is an objective measure of knowledge and skills, has robust psychometric properties, and contains scenarios relevant to occupational therapy. The AFT was however, adapted and tested in Australia and there were no published articles reporting its use in a low- to middle-income country context at the time of this study. Considering that EBP was a relatively new concept in occupational therapy in South Africa, and there was an indication that EBP knowledge and skills were likely to be low in this group [4], there was some concern about the clinical utility of the AFT in the South African context, and it was assumed that some modification would be needed.

The following research questions were framed: 1) what modifications are needed for the AFT to have clinical utility for use with South African occupational therapists at the early stages of EBP?, and 2) does the modified AFT have acceptable psychometric properties when used with novice South African occupational therapists before and after provision of education? The aims of this paper are therefore to: 1) describe amendments to the AFT for use with a cohort of South African occupational therapists, and 2) report the psychometric properties of the modified instrument (IRR and test-retest reliability, internal consistency and responsiveness as a measure of change) when used with South African occupational therapists.

## Methods

There were two phases in the study: 1) modifying the AFT, and 2) establishing the psychometric properties of the modified instrument.

### Instrumentation

The AFT was obtained from the developers who gave permission for its use. Version 2 was selected as the scenarios were more broadly applicable to occupational therapy practice in South Africa. The authors reviewed the AFT to judge its clinical utility, specifically completion and scoring times and applicability to the group being measured [16]. Evaluation of its applicability to occupational therapists in South Africa was based on anticipated EBP knowledge and skill levels as identified in the aforementioned South African occupational therapy survey [4].

Questions 1 to 3 in the AFT (see Table 2) test the ask and acquire steps of the EBP process which are important steps in getting started. While question 1 appears straight-forward it was anticipated that potential RCT participants may not be familiar with the Patient/Population, Intervention, Comparison, Outcome (PICO) format. Therefore the answer section was re-structured to indicate each PICO element in addition to the full question. Responses for questions 2 and 3 require some detail making them relatively time-consuming. The format of these questions was therefore changed to tick box answer options to reduce the time burden on respondents and raters (see Table 3). Similar modifications have been made to the Fresno Test to accommodate the learning needs of undergraduate health science students [13, 14].

**Table 2:** Evaluation of clinical utility of the AFT

Item	EBP step tested	Acceptable knowledge level	Acceptable completion time	Comment	Decision	Modification made
1. Write a focused question from one of two scenarios	Ask	Maybe	Yes	Straight forward and quick to complete but likely that participants may not know the PICO format	Modify	The answer section was structured to indicate the Population, Intervention, Comparative intervention and Outcome (PICO) format
2. Identify sources of information to answer clinical questions with their advantages and disadvantages	Acquire	Yes	No	Time-consuming to complete - requires identifying as many information sources as possible and listing advantages and disadvantages	Modify to reduce time burden	Format of question changed to tick boxes
3. Determine the appropriate study design to answer the question with a reason for the choice	Acquire	Maybe	Maybe	Open-ended question requiring study design and reason for choice	Modify	
4. Write a search strategy for the question with a rationale, and state how and why you could limit the search	Acquire	No	No	Consists of three parts and assumes familiarity in conducting MEDLINE searches. Considering that: 1) only 51 % of survey respondents had access to an academic library [4], 2) relatively few (22 %) used the internet to assist them in decision-making, and 3) just under half (42 %) lacked confidence in searching for literature, it was anticipated that many occupational therapists in South Africa may not be familiar with conducting MEDLINE searches. Furthermore, the questions were open-ended and thus demanding to complete	Remove	
5. Characteristics of a study that are used to determine its relevance to your practice	Appraise	No	No	Requires knowledge of research methods to critically appraise the relevance of a study and the validity, magnitude and significance of the findings. Considering the low confidence levels in research methods and critical appraisal identified in the survey [4] there was some concern that occupational therapist in South Africa would find these items overly challenging. If this induced participants to either omit items or fail to complete them to the best of their ability, the validity of the RCT results would be affected	Remove	
6. Characteristics of a study that may be used to determine if the findings are valid	Appraise	No	No			
7. Characteristics of the study findings that are used to determine their magnitude and significance	Appraise	No	No

**Table 3:**
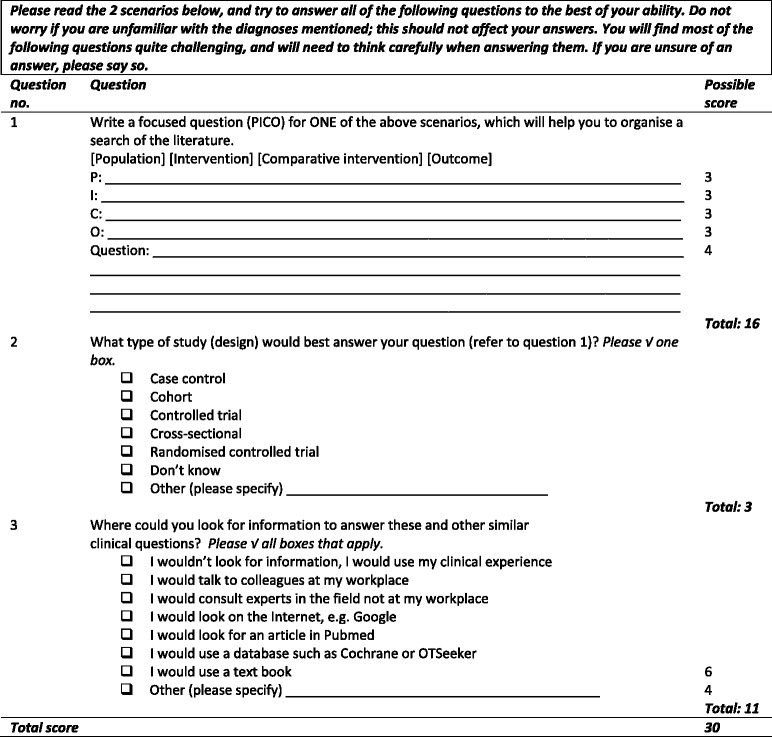
The Shortened Adapted Fresno Test of Competence in Evidence-based Practice (SAFT)

Question 4 assumes familiarity with MEDLINE searches. As just over 50 % of survey respondents had access to an academic library, few (22 %) used the internet to facilitate decision-making, and just under half (42 %) lacked confidence in searching for literature [4], it was assumed that many occupational therapists in South Africa may not be familiar with conducting MEDLINE searches. Questions 5 to 7 require sufficient knowledge of research methods to critically appraise the relevance of a study and the validity, magnitude and significance of the findings. Considering the low confidence levels in research methods and critical appraisal from the survey [4] there was some concern that potential RCT participants would find items 4–7 overly challenging. If this induced participants to either omit or fail to complete items to the best of their ability, the validity of the RCT results would be affected. These questions were therefore removed. While removal of four of the AFT items may have brought the content validity of the modified test into question, at the time there were no consensus guidelines to inform the choice of specific aspects of knowledge and skills that should be assessed [14]. Furthermore, it has been acknowledged that some health professionals may prefer to achieve a high level of EBP competence in only some domains, and that critical appraisal skills may not be essential for EBP [17]. The content of the instrument was thus designed to provide a realistic assessment of the key learning outcomes of the planned EBP training for participants with little or no exposure to EBP. Details of the evaluation and modifications to the AFT are shown in Table 2.

The modified version of the AFT was re-named the Shortened AFT (SAFT) and contained three items testing the first two steps of the EBP process (see Table 3 for details). The first author developed two new scenarios so the SAFT could be used to measure EBP knowledge and skills before and after an educational intervention (see Table 4). Prevalent health conditions treated by occupational therapists in South Africa were identified and scenarios written using the format in Versions 1 to 3 of the AFT. To accommodate as many areas of practice as possible, one mental health and one physical health condition was selected for the scenarios. A grading rubric was devised for the new scenarios (available on request from the first author). The grading rubric for the AFT developed by McCluskey and Bishop [8] was adapted to reflect the modifications and to ensure the scoring criteria were clear. The rubric was retained for question 1 (PICO) except that four additional points were awarded for writing out the complete question, and the scores for questions 2 and 3 were reduced to accommodate the new tick box format (see Table 5). The SAFT scoring was weighted slightly more heavily on step 1 as emphasis in the educational intervention was placed on getting the focus of the PICO questions clear in order to find the most appropriate article/s to inform practice. A comparison of the score weightings for the SAFT and AFT is available in Table 6.

**Table 4:** New scenarios for the final questionnaire

Scenario 1	Scenario 2
You work in an out-patients anxiety disorders clinic where you have been seeing a large number of young adults whose high levels of anxiety are affecting their productivity. They have been attending a support group but you have recently started wondering about the value of cognitive behavioural therapy in reducing anxiety levels and enabling them to cope more effectively with their everyday activities.	You have recently started receiving a number of referrals for people who have hypertension and cardiac problems. The focus of treatment until now has been provision of dietary advice and education. You are considering starting a stress management programme but would like to know whether this is likely to improve quality of life.

**Table 5:** Grading rubric for SAFT questions 2 and 3

Question no.	Items	Possible score
2. Type of study design	Randomised controlled trial	2
	Case control, cohort, controlled trial, cross-sectional	1
	Don’t know’	0
	Other - systematic review of randomised controlled trials	3
	Total	3
3. Where could you look for information …	‘I wouldn’t look for information, I would use my clinical experience’	0
	All remaining options – one point each	6
	Other:	
	• Other databases, e.g. Cinahl, PEDRo	1
	• Clinical guidelines	1
	• Professional organisations, e.g. South African Society of Hand Therapists	1
	• Conference proceedings	1
	• Continuing education courses	1
	Total	11

**Table 6:** Comparison of scoring proportions for each EBP step

Instrument	Ask	Acquire	Appraise
	No. of points (% of total)	No. of points (% of total)	No. of points (% of total)
SAFT	16 (53.3)	14 (46.7)	0 (0)
AFT	12 (7.7)	72 (46.2)	72 (46.2)

### Psychometric properties of the modified AFT

The psychometric properties of the SAFT were tested in two studies. Study 1 was a pilot to establish its reliability as the primary outcome measure for a pragmatic RCT, while Study 2 used data for the RCT intervention group to calculate the effect size.

### Participants

#### Study 1 – reliability

Convenience sampling was used to select participants who were as similar as possible to those for the proposed RCT. Occupational therapists in the Western Cape who met the following criteria were recruited:

- minimum of a 20 h working week;
- employed in:

- o private occupational therapy practices;
- o private hospitals;
- o hospitals funded by government departments other than health; and
- o Department of Health (DOH) facilities but were either not available at one or more of the key study points (baseline, intervention, 12-weeks post intervention), or declined to participate in the RCT.

Therapists who agreed to participate in the RCT were excluded. Participants were recruited by contacting the heads of identified occupational therapy departments and private practices to approach their staff. Department heads advised the first author of staff who agreed.

#### Study 2 - responsiveness

The population included all occupational therapists employed by the Western Cape DOH at the time of recruitment (*n* = 98). The only inclusion criterion was that participants had to be working at least 20 h per week. Those in management positions were included because of their role in putting structures and systems in place to support practitioners in their EBP endeavours [18]. For pragmatic reasons, therapists were excluded if they worked a distance of more than 90 min travelling time outside Cape Town, would be leaving the DOH before the end of the trial, or were taking leave during the time of the intervention, as this would compromise the outcome data.

As the primary outcome instrument (SAFT) was modified for the RCT, there was no data available to calculate the sample size prior to recruitment and data collection. For this reason, the maximum possible number of participants was recruited. Participants were recruited initially by linking with managers, and later through information sessions. Occupational therapy managers were asked to recruit staff in their departments and acted as the contact person for the researcher (first author) and research coordinator. Continuing professional development points were offered as an incentive to participate.

### Procedure

#### Study 1 – reliability

Hard copy questionnaires were delivered to all except two participants who received theirs via email. Participants completed the SAFT twice, 1 week apart. A week was considered sufficient to minimise the likelihood of participants recalling their previous responses or for EBP knowledge to have changed [19, 20]. Questionnaires were completed at participant’s convenience during the stipulated time period and collected after each completion. The two participants who received questionnaires by email returned them the same way. Completion of the questionnaires implied consent. The first author and a trained research assistant independently scored the SAFT at both time points (referred to as time 1 and time 2) using the grading rubric.

#### Study 2 - responsiveness

The SAFT was used as the primary outcome measure in a pragmatic RCT that aimed to establish the superiority of an interactive educational intervention (IE) over a didactic intervention (DE) in improving EBP knowledge at 12 weeks. Randomisation occurred in matched pairs by role (clinician or manager) and baseline SAFT score. The IE was multifaceted with two education sessions (of 4 and 2 h), emailed notes and telephonic or email reminders, while the DE was a single four-hour education session. Both interventions covered the five steps of the EBP process with an emphasis on using pre-appraised materials rather than critical appraisal. The IE included application exercises and discussion while the DE did not. Details of the RCT are available in a previous publication [3]. Baseline and final scores for the intervention group (IE) were used to calculate effect sizes to indicate the ability of the SAFT to detect change [19].

### Data management and analysis

The first author entered the data and double-checked entries for accuracy [21] against hard copies of the questionnaire. Data were entered by participant number with removal of identifying features to ensure confidentiality. Data were analysed with Statistical Package for the Social Sciences (SPSS) (Version 18).

#### Study 1 - reliability

Frequencies and proportions were calculated for demographic and practice profile data. Numerical data were checked for normality with the Shapiro-Wilk Test. As data were not normally distributed, medians and ranges were determined. Cronbach’s alpha was computed for internal consistency. As each SAFT item has a different score total, item scores were converted to percentages to determine Cronbach’s alpha. A value of 0.70 and above was regarded as satisfactory [22]. Intra-class correlation coefficients (ICCs) were calculated to assess test-retest reliability and IRR. A two-way random model for absolute agreement for single measures (type A,1) was used [23] with a recommended test value of 0.75 [24].

#### Study 2 – responsiveness

An intention-to-treat analysis was performed in which participants who did not complete the SAFT at 12-weeks were regarded as being unchanged (baseline data were carried forward as their 12-week scores) [25]. As the Shapiro-Wilk Test revealed that the data were not normally distributed, non-parametric statistics were used. Medians and ranges were calculated for the IE group for individual SAFT items and total scores for baseline and 12-week data. Median change scores and ranges were also determined. The Wilcoxon Signed Rank Test was used to determine whether there were significant differences in individual items and total SAFT scores from baseline to 12 weeks. Effect sizes for the Wilcoxon Signed Rank Test were calculated by dividing the Z-score by the square root of the number of observations. Cohen’s benchmarks [26] were used to interpret effect sizes. Based on a previous study where a 10 % improvement in the mean total AFT score post intervention was regarded as educationally important [8], an improvement of at least 3.0 points (10 % of a total possible score of 30 points on the SAFT) was anticipated in the IE group in the RCT.

### Ethical approval and considerations

Ethical approval was obtained from the Health Sciences Faculty Human Research Ethics Committee, University of Cape Town (REC REF: 259/2006) and the Western Cape Provincial Department of Health (Ref. 19/18/RP37/2008). Letters were sent to medical superintendents and senior managers at participating facilities to inform them about the study and obtain their support. Questionnaires were numbered to ensure anonymity and confidentiality. Responses were treated confidentially and only the first author had access to the list linking questionnaire numbers with participant’s names.

## Results

### Study 1 - reliability of the shortened AFT (SAFT)

Of the 26 occupational therapists who volunteered to participate, 21 completed the questionnaire on two occasions. Completion of the questionnaires was regarded as having given consent. Five were excluded from the analysis because they were on annual leave at one completion time (*n* = 4) or completed the second questionnaire early due to going on annual leave (*n* = 1). The median duration between completion times was 7.0 (min-max: 7.0–49.0) days.

### Sample demographics

All participants were female and worked in urban areas. Many worked in tertiary settings (14/21, 66.7 %). Medians for age and experience were 29.0 (min-max: 23.0–57.0) years and 7.0 (min-max: 1.0–29.0) years respectively. Most had a bachelors’ degree in occupational therapy (19/21, 90.5 %) with two having postgraduate occupational therapy qualifications.

### Internal consistency

The internal consistency of the SAFT (baseline version) was satisfactory for time 1 scores (α = 0.70). Removal of individual items did not improve the internal consistency of the scale.

### Inter-rater reliability

IRR was excellent for individual SAFT items (ICCs ranged from 0.89 to 1.0) and total scores for both completion times (Time 1 scores: ICC = 0.995, 95 % CI: 0.99–0.998; Time 2: ICC = 0.99, 95 % CI: 0.97–0.995). See Table 7 for details.

**Table 7:** Inter-rater reliability of the SAFT for times 1 and 2 (*n* = 21)

Item (possible score)	ICC (95 % CI)	Strength of agreement^a^
Time 1		
PICO score (16)	0.99 (0.98–0.997)	Excellent
Study design score (3)	1.00	Excellent
Source score (11)	1.00	Excellent
Total score (30)	0.995 (0.99–0.998)	Excellent
Time 2		
PICO score (16)	0.99 (0.97–0.995)	Excellent
Study design score (3)	1.00	Excellent
Source score (11)	0.89 (0.76–0.96)	Excellent
Total score (30)	0.99 (0.97–0.995)	Excellent

^a^ICC values were rated as poor (<0.40), fair to moderate (0.41–0.59), good (0.60–0.74) or excellent (>0.75) [24]

### Test-retest reliability

Test-retest reliability was good to excellent for individual items (ICCs ranged from 0.71 to 0.96) and excellent for total scores (ICC = 0.95, 95 % CI: 0.88–0.98). Refer to Table 8 for details.

**Table 8:** Test-retest reliability for the SAFT (*n* = 21)

Item (possible score)	ICC (95 % CI)	Strength of agreement^a^
1. PICO score (16)	0.96 (0.91–0.99)	Excellent
2. Study design score (3)	0.87 (0.71–0.95)	Excellent
3. Source score (11)	0.71 (0.41–0.87)	Good
Total score (30)	0.95 (0.88–0.98)	Excellent

^a^ICC values were rated as poor (<0.40), fair to moderate (0.41–0.59), good (0.60–0.74) or excellent (>0.75) [24]

### Study 2 - responsiveness

The RCT sample consisted of occupational therapists employed in four Western Cape DOH district municipalities (*n* = 58) who provided written informed consent. Twenty-eight were randomly allocated to the IE group and completed the SAFT at baseline and 12 weeks after attending an educational intervention. None of the participants were involved in Study 1.

### Sample demographics

Medians for age and experience in the IE group were 28.0 (min-max: 22.0–50.0) years and 5.5 (min-max: 0.5–31.0) years respectively. Most participants were female (26/28; 92.9 %), had undergraduate qualifications (24/28; 85.7 %) and worked in tertiary facilities (10/28; 35.7 %) in urban areas (23/28; 82.1 %). The median duration between the intervention and completing the final questionnaire was 13.0 (min-max: 11.0–22.0) weeks.

### Changes in knowledge between baseline and 12 weeks

Median total SAFT scores increased in the IE group from 14.0 points at baseline (min-max: 2.0–23.0) to 21.0 (min-max: 2.0–25.0) points post intervention. The Wilcoxon Signed Rank Test showed this improvement in knowledge to be significant (*Z* = −4.078, *p* < 0.001) with a moderate effect size (*r* = 0.55) (refer to Table 9).

**Table 9:** Responsiveness of the SAFT (*n* = 28)

Question (score)	Median at baseline (Min-max)	Median at 12-weeks (Min-max)	Median change (Min-max)	Z (*p*-value)*	Effect size (r)	Interpretation^a^
PICO (16)	8.0 (0.0–16.0)	16.0 (0.0–16.0)	5.0 (0.0–16.0)	−4.11-(**<0.001**)	0.55	Moderate
Study design (3)	0.0 (0.0–2.0)	1.0 (0.0–2.0)	0.0 (−1.0–2.0)	−2.39 (**0.017**)	0.32	Small
Sources of information (11)	4.0 (1.0–6.0)	5.0 (2.0–8.0)	0.0 (−2.0–5.0)	−1.66 (0.097)	0.22	Small
Total score (30)	14.0 (2.0–23.0)	21.0 (2.0–25.0)	5.5 (−2.0–19.0)	−4.08 (**0.001**)	0.55	Moderate

*Significant *p*-values are indicated in bold

^a^Based on Cohen’s [26] values for interpreting effect sizes: > 0.80 = large; 0.50–0.80 = moderate; < 0.50 = small

## Discussion

Despite the SAFT being considerably shorter and simplified in its structure and content, it demonstrated comparable psychometric properties to the AFT. In particular, IRR for the SAFT (Time 1 scores: ICC = 0.995, 95 % CI: 0.99–0.998; Time 2: ICC = 0.99, 95 % CI: 0.97–0.995) is equivalent to that obtained by McCluskey and Bishop [8] for total AFT scores (Version 2) (ICC = 0.91, 95 % CI: 0.69–0.98). Furthermore, the narrow 95 % confidence intervals (CIs) for SAFT individual items and total scores indicate the high precision in scoring between raters [16]. The excellent scores for test-retest reliability indicate that the SAFT is stable in the absence of change and thus able to describe EBP knowledge and skills [16, 19, 20, 27].

The 23 % increase in total SAFT score between baseline and 12 weeks is substantially higher than those of Novak (6.5 % increase) [10], McCluskey (13.2 %) [8] and Crabtree (14.6 %) [11], but compares favourably with Lizarondo (21.0 %) [9]. This may be due to the focus only on the first two steps of the EBP process. The SAFT was responsive to measuring change in knowledge and skills with a moderate effect size for the total score (*r* = 0.6). This parallels the effect sizes reported by McCluskey and Bishop (*d* = 0.8) [8] and Novak and McIntyre (*d* = 0.6) [10]. Question 1 for the SAFT and the AFT showed a moderate effect size (*r* = 0.6 and *d* = 0.8 respectively). As the scenarios, wording and scoring of the PICO question were largely unchanged this similarity is not surprising. On the other hand, questions 2 (study design) and 3 (sources where information may be found) of the SAFT had small effect sizes (0.3 and 0.2 respectively), while question 2 of the AFT had a small effect size (*d* = 0.1), but question 3 was large (*d* = 0.9). Test-retest reliability was also the lowest for question 3 of the SAFT with wide 95 % CIs (ICC = 0.71, 95 % CI: 0.41-0.87). This may have been due to the wording of the item - participants were required to select the sources of evidence they would use rather than all the possible sources they could use to answer the PICO question. This may either have resulted in participants changing their minds for the second completion or reporting only the sources they would use. It is therefore recommended that the wording of this item be changed as shown in Table 3. The wide 95 % CIs may also have been due to the small sample size.

Removal of four of the AFT items is likely to have influenced the content validity of the SAFT as it measures only the first two steps of the EBP process. However, the AFT does not measure the entire EBP process either having been critiqued for its inability to measure the apply step [11]. It has been suggested that the competencies needed for EBP may differ across health professions [14]. This assertion led to a modification of the AFT for physiotherapists (the K-REC) which similarly focuses on novice learners and clinicians and covers only the first three steps of the EBP process [14]. These authors contended that instruments may be required for specific purposes and may therefore not need to assess all five steps of the EBP process. It has been argued that proficiency in critical appraisal may not be an essential pre-requisite for EBP, and thus it may not be necessary to evaluate this step of the EBP process [17]. The intervention tested in the pragmatic RCT reported in this paper and elsewhere [3] focussed on using pre-appraised sources rather than critical appraisal which justifies the focus on steps 1 and 2 and the exclusion of step 3.

The time-consuming nature of completion and scoring has been identified as a short-coming of both the original Fresno Test [13] and the AFT [14]. The revised response options in the SAFT resemble those of the K-REC which similarly uses tick boxes and also simplified the scoring substantially [14]. Although the SAFT assesses only the first two steps of the EBP process, it is quick to complete and score and is useful for therapists in the early stages of learning about EBP where knowledge and skills are limited. Therefore, it may be more applicable in contexts where there has been limited EBP exposure whereas the AFT is recommended for groups who have progressed beyond the early stages of EBP as it tests more advanced search and critical appraisal skills.

A strength of this study is the use of the SAFT in a RCT. Few studies have employed a comparison group to contribute validation data to EBP measurement instruments [28]. Furthermore, this is one of few studies to be conducted in a middle-income country which contributes a different perspective to the existing literature [28]. Although this study reports the use of the SAFT with occupational therapists, the instrument may easily be adapted for other rehabilitation professions by modifying the current scenarios or writing new profession-specific or multi-professional scenarios and revising the grading rubrics.

A limitation of this study is the small sample size and the inclusion of only occupational therapists in the public health sector in one area of South Africa. It is therefore unclear whether the findings apply to occupational therapists working in other sectors (for example, private practice, or government departments, such as education) and provinces in South Africa. It is furthermore unclear to what extent the findings may be generalised to occupational therapists in other low- and middle-income countries or to other health professional groups.

There is a paucity of research on instruments to measure EBP knowledge and skills in contexts where health professionals have had limited or no exposure to EBP training. Instruments such as the AFT, intended for novice evidence-based practitioners, are too advanced and time intensive. The content of EBP measurement instruments needs to match the knowledge and skill levels of the target group and the outcomes of the training to be provided. This requires a consensus process to establish the levels of knowledge and skill required by professionals from those with no previous EBP training right up to those who are more advanced. Future research should test the SAFT with a wider population of occupational therapists and other health professions to evaluate transferability. Investigation into valid and reliable instruments in contexts where EBP is in its early stages would build the limited current evidence base in these settings, and inform the development of additional instruments to test all five steps of the EBP process.

## Conclusion

The SAFT demonstrated its validity for detecting differences between two groups in EBP knowledge related to the first two steps of the EBP process. It also has excellent IRR and test-retest reliability. Its psychometric properties compare favourably with the AFT suggesting that it offers another option for researchers conducting studies in contexts where EBP is less advanced. As an instrument that is also quick and easy to complete and score, it may be a useful research tool in contexts where EBP knowledge levels are low and time is limited.

## Abbreviations

AFT: Adapted Fresno Test of Competence in Evidence-based Practice
CIs: Confidence intervals
DE: Didactic educational intervention
DOH: Department of Health
EBM: Evidence-based Medicine
EBP: Evidence-based practice
KAB: Knowledge, attitude and behavior
K-REC: Knowledge of research evidence competencies
ICC: Intra-class correlation coefficient
IE: Interactive educational intervention
IRR: Inter-rater reliability
RCT: Randomised controlled trial
SAFT: Shortened Adapted Fresno Test
